# Supplementary material for: Role of Micronutrient Supplementation in Promoting Cognitive Healthy Aging in Latin America: Evidence-Based Consensus Statement
Source: Nutrients. 2025 Aug 2;17(15):2545. doi: 10.3390/nu17152545 (PMC12348371; doi:10.3390/nu17152545)
Supplement: Supplementary file 1 [file nutrients-17-02545-s001.zip › nutrients-3786601-supplementary.pdf]

SUPPLEMENTARY FIGURES

.

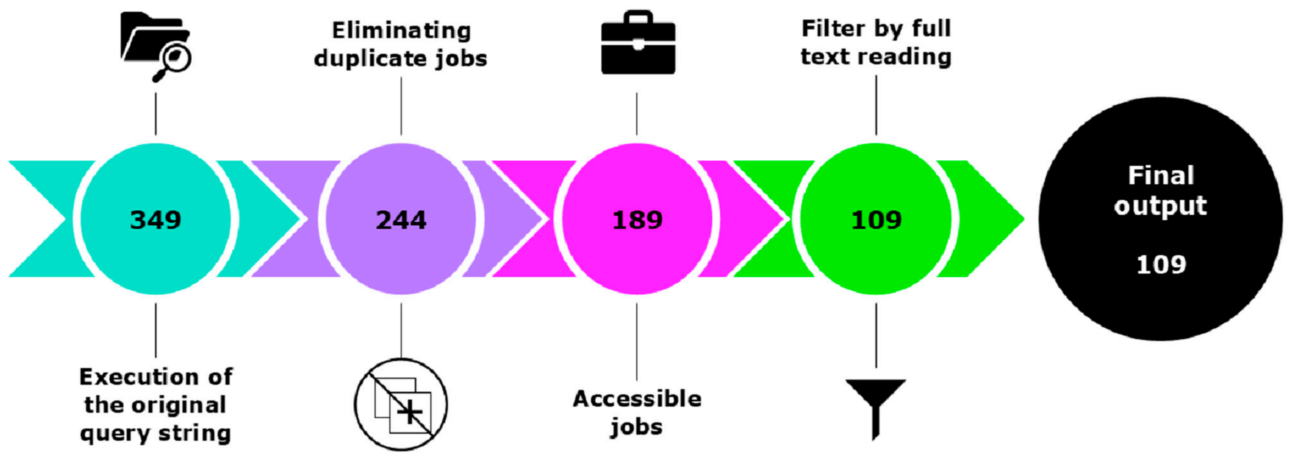

Supplementary Figure S1: Search and selection process of articles

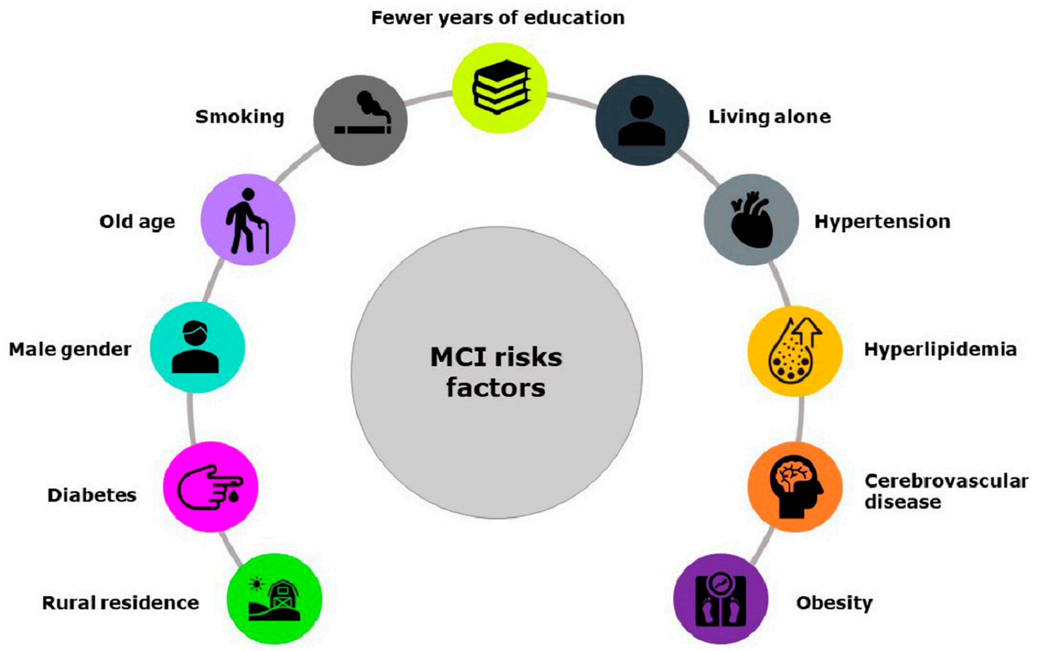

Supplementary Figure S2: Risk factors of Mild Cognitive Impairment (MCI) [63]

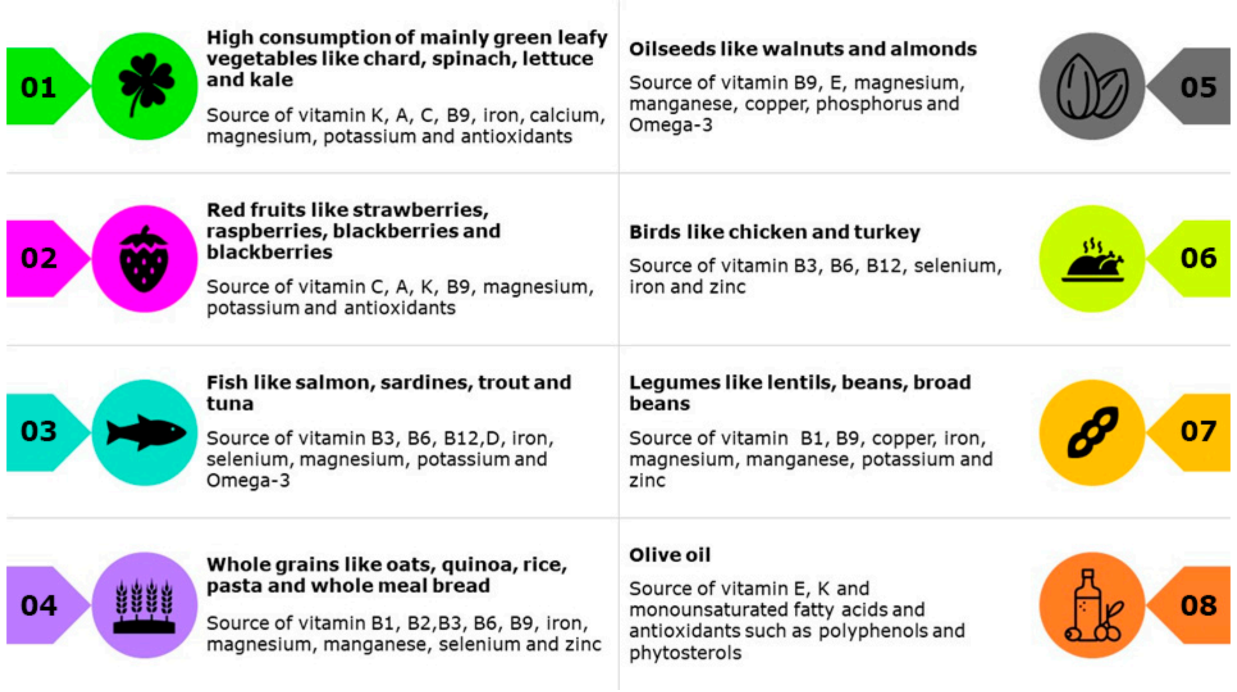

Supplementary Figure S3: Dietary pattern of the MIND Diet [35]

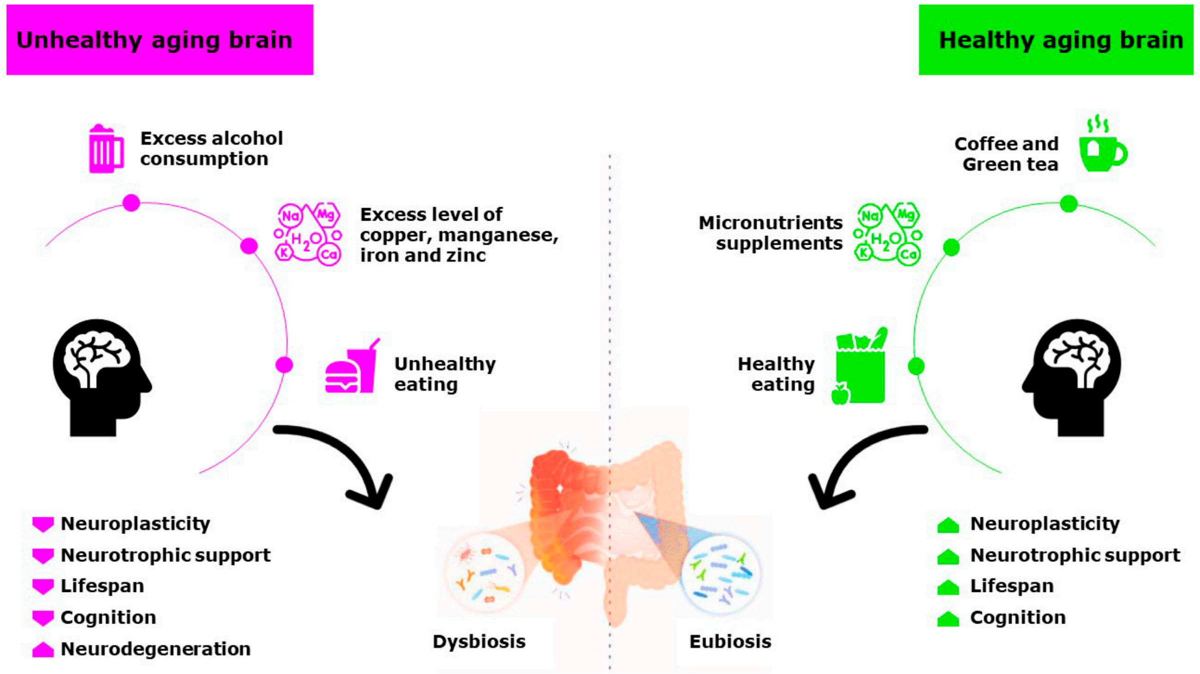

Supplementary Figure S4: The contribution of diets to brain health during aging [58]

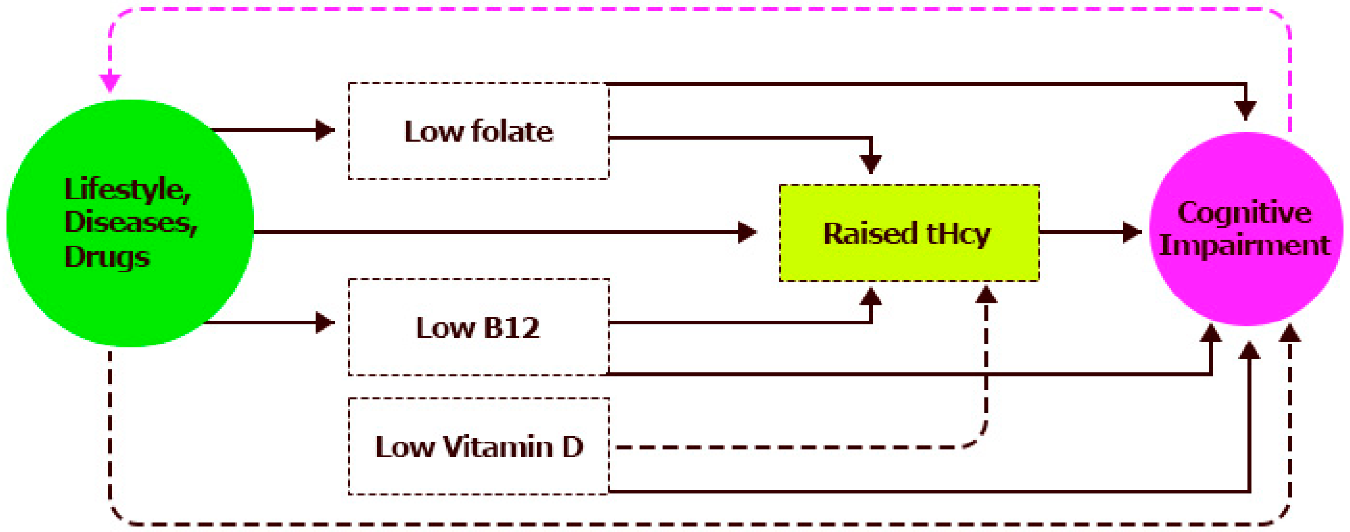

Supplementary Figure S5: Specific interactions of vitamins with homocysteine in brain [63]

SUPPLEMENTARY TABLES

Supplementary Table S1: Search Strategy for Embase

Database	Search Terms	Inclusion Criteria	Exclusion Criteria
Embase	**Vitamins/Minerals Terms:**<br>- vitamin$ adj5 dietary:ab ,ti<br>- vitamin$ adj5 supplement$:ab ,ti<br>- mineral$ adj5 dietary:ab ,ti<br>- mineral$ adj5 supplement$:ab ,ti<br>- multivitamin$:ab ,ti<br>- multi-vitamin$:ab ,ti<br>- multimineral$:ab ,ti<br>- multi-mineral$:ab ,ti<br>**Cognitive Terms:**<br>- Cogni*:ti ,ab<br>- mild cognitive impairment:ti ,ab<br>- dementia:ti ,ab<br>- vascular dementia:ti ,ab<br>- Alzheimer:ti ,ab<br>**Geographic Terms:**<br>- Latin America:ti ,ab<br>- South America:ti ,ab<br>- Caribbean:ti ,ab<br>- Individual countries (e.g., Argentina, Brazil, Mexico, etc.):ti ,ab.	**Population:**<br>Studies involving older adults in Latin America, South America, or the Caribbean.<br>**Intervention/Exposure:**<br>Multivitamin and mineral (MVM) supplementation, Dietary vitamins and minerals relevant to cognitive function.<br>**Study Designs:**<br>Randomized controlled trials (RCTs), Cross-sectional studies, Systematic reviews and meta-analyses, reviews<br>**Outcomes:**<br>Cognitive function assessments, memory performance, cognitive decline measures, nutrient deficiencies related to cognitive health.	**Population:**<br>Studies focusing on children or non-Latin America<br>**Study Designs:**<br>Editorials, commentaries, and letters (lack scientific rigor).<br>Studies with insufficient data, such as those without clear outcome measures or inadequate reporting.

Supplementary Table S2: Search Strategy for PubMed

**Database**	**Search Terms**	**Inclusion Criteria**	**Exclusion Criteria**
PubMed	**Vitamins/Minerals Terms:** (Multivitamin* OR "Multi-vitamin*" OR multimineral* OR "multi-mineral*" OR Iron OR zinc OR magnesium OR Vitamin* OR mineral* OR "dietary supplement*")title/abstract<br>**Cognitive Terms:**<br>(Cogni* OR "mild cognitive impairment" OR "dementia" OR "vascular dementia" OR "Alzheimer")title/abstract<br>**Geographic Terms:**<br>("Latin America" OR "South America" OR Caribbean OR Argentina OR Bolivia OR Brazil OR Chile OR Colombia OR "Costa Rica" OR Cuba OR Ecuador OR "El Salvador" OR Guatemala OR Haiti OR Honduras OR Mexico OR Nicaragua OR Panama OR Paraguay OR Peru OR "Dominican Republic" OR Uruguay OR Venezuela OR Jamaica OR "Trinidad and Tobago" OR Guyana or Suriname OR Belize OR Bahamas OR Barbados OR "Saint Lucia" OR Grenada OR "St. Vincent and Grenadines" OR "Antigua and Barbuda" OR Dominica OR "Saint Kitts and Nevis")title/abstract	**Population:**<br>Studies involving older adults in Latin America, South America, or the Caribbean.<br>**Intervention/Exposure:**<br>Multivitamin and mineral (MVM) supplementation, Dietary vitamins and minerals relevant to cognitive function.<br>**Study Designs:**<br>Randomized controlled trials (RCTs), Cross-sectional studies, Systematic reviews and meta-analyses, reviews<br>**Outcomes:**<br>Cognitive function assessments, memory performance, cognitive decline measures, nutrient deficiencies related to cognitive health.	**Population:**<br>Studies focusing on children or non-Latin America<br>**Study Designs:**<br>Editorials, commentaries, and letters (lack scientific rigor).<br>Studies with insufficient data, such as those without clear outcome measures or inadequate reporting.

Supplementary Table S3: Insufficiency/Deficiency of Micronutrients in Elderly Latin American Population

Micronutrients Insufficiency/ Deficiency	Brazil	Argentina	Chile	Costa Rica	Ecuador	Mexico	Colombia	Peru
**Vitamin B12**	Martinho 2015 [67]	Cavagnari 2021	Olivares 2000 [74] S´anchez 2010 [75] Castillo-Lancellotti 2013 [76]	Dinamarca-Montecinos 2022 [77] Ministerio de Salud 2012 [78]	Hamer 2009 [68] Vinueza Veloz 2022 [79]	Pereda 2006 [80] De la Cruz-Gongora 2021 [81]	Cavagnari 2021	Diaz 2021 [82]
**Folate**	-	-	Hirsch 2002 [62] Olivares 2000 [74] Castillo-Lancellotti 2013[76]	Ministerio de Salud 2012 [78]	Hamer 2009 [68]	Pereda 2006 [80] De la Cruz-G´ongora 2021 [81]	-	Diaz 2021 [82]
**Vitamin D**	Pereira-Santos 2018 [83] Lima-Costa 2020 [84]	Cavagnari 2021, Puche 2015	Carrasco 2014 [85] , Ministerio de Salud 2020 [78]	-	Hamer 2009 [68] Orces 2015 [86]	Carrillo-Vega 2017 [87] , Carrazco-Pen˜a 2022 [88] Mendoza-Garc´es 2021 [89]	Amaya-Montoya 2021, Cavagnari 2021	-

Micronutrients Insufficiency/ Deficiency	Brazil	Argentina	Chile	Costa Rica	Ecuador	Mexico	Colombia	Peru
						Pedroza-Tob´ıas2015		
**Vitamin A**	-	Cavagnari 2021	Olivares 2000 [74]	-	Hamer 2009 [68]	Gonz´alez 2021[90]	Cavagnari 2021	-
**Vitamin C**	-	Cavagnari 2021	-	-	Hamer 2009 [68]		Cavagnari 2021	-
**Vitamin E**	-	-	-	-	Hamer 2009 [68] Pedroza-Tob´ıas2015		-	-
**Iron**	Sales 2021[91]	Cavagnari 2021	-	-	Hamer 2009 [68]	Contreras-Manzano 2015 [92]<br>Cruz-G´ongora 2021 [81]<br>Rivera-Pasquel 2021 [93] ´anchez-Pimienta 2012	Cavagnari 2021	-
**Zinc**	Marchetti 2022 [94]	Cavagnari 2021	-	-	Hamer 2009 [68]		Cavagnari 2021	-

*Expert recommendation for insufficiency/deficiency of micronutrients in elderly Latin American population was; Strongly Agree:3 and Agree:6

Supplementary Table S4: Association between vitamin B supplementation and cognitive function

Micronutrient Supple mentation	Citation	Intervention	Results
Vitamin B6	Zhang C et al. 2022 [95]	Magtein: 400 mg®<br>Vitamin D_3: 80 IU<br>Vitamin C: 12 mg<br>Vitamin B6: 4 mg.<br>Phosphatidylserine 50 mg<br>Total: 2 g/person/day	Magtein®PS: Significant improvement in memory and cognition in healthy Chinese adults, aged 18 to 65 years old ($p < 0.001$).
Vitamin B1	Gibson GE et al. 2020 [96]	Benfotiamine treatment (300 mg/day twice a day) versus placebo group.	Benfotiamine Group vs. Placebo Group: 43% lower increase in ADAS-Cog Scores and 77% less worsening in CDR ($p = 0.034$) in Benfotiamine Group.
Folate/Vitamin B12	Ma F L et al. 2019 [97]	Four treatment groups: 800 µg FA only, 25 µg vitamin B12 only, FA, and vitamin B12 supplementation or control group.	Supplementation with FA and Vitamin B12: Significant improvements in FSIQ ($d = 0.169$, $p = 0.024$), verbal IQ ($d = 0.146$, $p = 0.033$), Information ($d = 0.172$, $p = 0.019$), and Digit Span Scores.
Folate/Vit. B12	Kwok T et al. 2020 [98]	MCI patients administered. 500 µg methylcobalamin and 400 µg FA orally once daily.	Supplementation with Vitamin B12 and FA: No reduction in cognitive decline in older individuals with MCI and elevated serum homocysteine.

*Expert panel recommendation for the association of vitamin B supplementation and cognitive function was; Strongly Agree:3 and Agree:6

ADAS-Cog: Alzheimer's Disease Assessment Scale-Cognitive, CDR: Clinical Dementia Rating, FSIQ: Full Scale IQ, FA: Folic Acid, MCI: Mild Cognitive Impairment

Supplementary Table S5: Association between vitamin D supplementation and cognitive function

Micronutrient Supplementation	Citation	Intervention	Results
Vitamin D	Jia J et al. 2019 [99]	Patients received 800 IU/day of vitamin D	The FSIQ and cognitive test score were significantly higher in the intervention group than in the control group ($p < 0.001$).
	Bischoff-Ferrari HA et al. 2020 [100]	2000 IU/day of vitamin D_3, 1 g/day of omega-3 strength-training exercise program	Among adults aged 70 years or older, treatment with vitamin D_3, omega-3, or a strength-training exercise program did not result in statistically significant differences in improving cognitive function.
	Yang T et al. 2020 [101]	800 IU/day of vitamin D_3	The ANOVA showed improvements in the FSIQ, information, digit span, vocabulary, block design, and picture arrangement scores in the vitamin D group over the placebo group ($p < 0.001$).
	Castle M et al. 2020 [102]	Vitamin D_3 supplementation (600, 2000, or 4000 IU/day)	The CANTAB test results indicated that the 2000 IU/d group, when compared to other groups, performed better in visual and working memory and new learning parameters ($p < 0.05$).
	Ghaderi A et al. 2020 [103]	Administration of either 50,000 IU Vitamin D supplements (n = 32) or placebo (n = 32) every 2 weeks	Subjects who were administered vitamin D, had a significant reduction in IGT (β −6.25; 95% CI, −8.60 to −3.90; $p < 0.001$),<br>and significant increases in VFT (β 2.82; 95% CI, 0.78–4.86; p = 0.007), immediate LM (β 1. 32; 95% CI, 0.27–2.37; p = 0.01).
	Hu J et al. 2018 [104]	Administration of 800 IU/day of vitamin D	The mean scores of information, DGS, vocabulary, block design and picture arrangement tests in the vitamin D_3 group were significantly higher than that in the placebo group both before and after adjustment.
	Beauchet O et al. 2019 [105]	Fortified yogurt (400 IU vitamin D and 800 mg calcium)	Fortified yogurts with vitamin D and calcium maintained global cognitive performance (MMSE score (p = 0.022) which was decreased in the control group.

Micronutrient Supplementation	Citation	Intervention	Results

*Expert panel recommendation for the association of vitamin D supplementation and cognitive function was; Strongly Agree:3 and Agree:6

ANOVA: Analysis of Variance, CANTAB: The Cambridge Neuropsychological Test Automated Battery, DGS: Digit Span, FSIQ: Full Scale IQ, IGT: Impaired Glucose Tolerance, LM: Logical Memory, MMSE: Mini-Mental State Examination, VFT: Verbal Fluency Test
